# The DN-PUBLIC framework for enhanced oral healthcare precision: a public health strategy for dynamic navigation integration—a narrative review

**DOI:** 10.3389/froh.2025.1598206

**Published:** 2025-05-13

**Authors:** Ashwini Bhalerao, Vaibhav Kumar

**Affiliations:** ^1^ Department of Oral and Maxillofacial Surgery, Saveetha Dental College, Chennai, India; ^2^ Department of Public Health Dentistry, Dr GD Pol Foundation YMT Dental College and Hospital (Affiliated to Maharashtra University of Health Sciences), Navi Mumbai, India

**Keywords:** dynamic navigation, dental implants, oral health equity, digital dentistry, public health strategy, minimally invasive techniques

## Abstract

Oral health disparities remain a pressing global concern, especially in communities with limited access to specialized dental care. Implant dentistry, while transformative for tooth replacement, often relies on techniques that can be imprecise, operator-dependent, and prone to complications. Dynamic Navigation (DN), a real-time computer-assisted technology, offers a promising solution by enhancing accuracy, reducing errors, and supporting minimally invasive procedures. This narrative review explores how DN can improve clinical precision, reduce surgical complications, and make implantology more accessible and cost-effective. It introduces the DN-PUBLIC framework—a strategic, public health-focused approach for integrating DN into broader healthcare systems, with a strong alignment to the United Nations Sustainable Development Goals (SDGs).A comprehensive review of current literature was conducted, assessing DN's impact on surgical safety, recovery outcomes, cost-efficiency, and its growing role in dental education. The findings highlight that DN significantly improves implant placement accuracy and reduces risks such as nerve injury or misalignment. By allowing for flapless procedures and better soft tissue preservation, DN leads to quicker recovery and greater patient comfort. Beyond clinical outcomes, DN enables general practitioners to perform complex procedures more confidently, expanding access to quality care in underserved regions. Economic analyses also suggest reduced operative time, fewer complications, and lower healthcare costs. In conclusion, DN has the potential to transform public oral health by improving outcomes, training, and access. The DN-PUBLIC framework offers a clear roadmap to guide ethical, inclusive, and sustainable integration of DN technology in dental practice worldwide.

## 1 Introduction

Oral health disparities represent a significant global public health challenge, disproportionately affecting millions of individuals due to a combination of socioeconomic inequalities, geographical barriers, and limited access to specialized dental care. These disparities often result in untreated tooth loss, leading to functional, aesthetic, and psychological burdens that can significantly impact an individual's quality of life. Among the most affected populations are those suffering from edentulism, severe maxillary atrophy, and patients requiring post-maxillectomy rehabilitation following oncologic resections or traumatic injuries (1).

Dental implants have emerged as a transformative intervention, offering a long-term solution for tooth loss by restoring masticatory function, improving speech, and enhancing overall oral health. Conventional implantological procedures create multiple difficulties because they result in reduced accuracy together with anticipated complications and operator-dependent outcome uncertainties because of complex anatomical factors. Therapeutic implant placement when done freehand usually results in trajectory plan deviations that create osseointegration risks and nerve and sinus perforations that require additional surgical repairs. The need for extensive bone grafting procedures becomes a major challenge in treatment when severe bone loss occurs because it adds complications and raises treatment costs (2).

The state-of-the-art technology Dynamic Navigation (DN) has transformed implantology by solving previous operational issues. The integration of CBCT high-resolution cone-beam computed tomography in DN enables intraoperative tracking along with visualization to precisely guide real-time implant placement. The modern technology makes surgeries more precise while cutting down errors and decreasing sole reliance on operators to provide improved dental implant stability over time. The technology provides ongoing feedback combined with intraoperative adjustments to enable minimally invasive procedures which lowers patient postoperative risks and speeds up patient recovery and improves final treatment success (3).

DN technology becomes highly significant in areas where implantologist specialists are scarce because it creates procedural standardisation with improved accuracy which leads to reduced disparities in implant success rates. DN integration into standard clinical practice shows strong potential to enhance healthcare delivery for implantology especially toward underserved populations by offering better accessibility and cost-effectiveness and superior patient outcomes (4).

DN systems control implant misalignment which decreases surgical complications hence decreases the number of hospital operations requiring restorative procedures and reduces overall healthcare spending. This review explores how Digital Navigation technology can revitalise public health services through enhanced access and procedural safety and reduced expenses and training capabilities in dental practise (5–9).

## 2 Methodology

This narrative evaluation targeted the assessment of Dynamic Navigation (DN) usefulness for public health through dental implantology when addressing differential access to oral precision medical services. The SANRA (Scale for the Assessment of Narrative Review Articles) criteria provided guidance for selecting relevant literature along with determining this review's structure since they focus on non-systematic reviews with clarity and scientific rigour and relevance.

The authors performed an extensive review of published and grey literature from January 2023 through March 2024. The research utilised PubMed alongside Scopus and Web of Science and Google Scholar databases for database consultations. The search was iterative and manually refined over time, with the following keywords and keyword combinations used: “dynamic navigation,” “real-time implant placement,” “dental implants,” “surgical accuracy in implantology,” “oral health disparities,” “digital dentistry,” and “cost-effectiveness of DN systems.” Boolean operators were employed to expand or narrow down results as necessary, depending on the scope of the article being screened.

Articles were screened based on their titles and abstracts, and full texts were reviewed when they aligned with the review's objectives. No strict inclusion or exclusion criteria were applied, as is typical in systematic reviews, but preference was given to studies that (1) investigated the clinical outcomes of DN-guided implant procedures, (2) discussed its implementation or cost-effectiveness, and (3) addressed educational or training aspects related to the use of navigation systems in dental practice. The search included original research articles, case series, narrative and systematic reviews, clinical trials, and select conference abstracts if the findings were relevant and not available elsewhere.

Manual cross-referencing of bibliographies was also carried out, especially in more recent reviews and meta-analyses, to identify additional primary studies or reports. Some grey literature and preprints were consulted when the topic was emerging or where peer-reviewed data were limited, particularly in the context of public health or accessibility discussions.

All references were managed manually using a combination of note-taking and spreadsheet documentation to categorize sources by topic area (accuracy, accessibility, safety, training, cost, etc.). No formal bias assessment tools were used due to the narrative nature of this work; however, potential limitations, conflicting findings, and areas of weak evidence were noted and are discussed in a separate section.

This methodology was chosen to allow for a broad, flexible, and evolving synthesis of information, given that the integration of DN into public health-focused oral healthcare remains a relatively new and dynamic topic. The review does not claim to be exhaustive but aims to provide a thoughtful, evidence-informed perspective on the emerging role of DN systems in improving both clinical precision and healthcare equity.

### 2.1 The public health significance of dynamic navigation

#### 2.2.1 Addressing disparities in access to high-quality dental care

People in low-resource communities face difficulties when accessing specialized dental surgery services. DN enables the standardisation of implant placement accuracy which enables general practitioners to conduct complex procedures with the same level of expertise as specialists (10). Research indicates that Digital Navegation improves both safety and predictability of treatment results for less-skilled operators which creates wider healthcare opportunities for improved public health outcomes (11, 12).

#### 2.2.2 Enhancing surgical precision and reducing complications

The technique of freehand implant placement causes deviations in angulation and depth which results in nerve damage and implant failure and peri-implantitis according to (13). DN offers a solution to minimise these complications through its ability to display procedural data in real time thus enabling clinicians to maintain accurate implant placement (14). Zygomatic implant placement using DN guidance produces better accuracy than traditional methods because it results in significantly smaller deviations between planned and achieved positions (5, 7, 15).

#### 2.2.3 Minimizing postoperative morbidity and improving recovery outcomes

The recovery process and patient life quality suffer from complications including pain and swelling together with sensory disturbances after surgery. Patient healing becomes quicker because DN allows minimally invasive procedures without needing surgical flaps while maintaining tissue health thus minimizing complications (16). The utilisation of DN-assisted flapless implant placement for surgery resulted in lower postoperative pain alongside reduced swelling and decreased need for analgesics compared to traditional implant placement methods according to research findings (6).

#### 2.2.4 Cost-Effectiveness and resource optimization in dental healthcare

Public health policy requires healthcare cost burden to be a key factor of consideration. DN generates financial savings by minimising surgical mistakes and shortening operative time as well as minimising implant failure rates and postoperative surgeries (17). The study by Bhalerao et al. proved that DN-guided flapless implant placement delivers substantial economic advantages which makes it suitable for both private and public healthcare centres (7).

#### 2.2.5 Advancing clinical training and education

DN serves as a revolutionary educational technology which provides instantaneous feedback and exact learning conditions for dental education. Training programmes that incorporate DN technology enable new practitioners to learn implantology at higher competency levels thus decreasing the complexity of zygomatic implant placement (18, 19). DN plays a critical role in skill development because it offers standardized training which leads to improved expert competence in advanced implantology according to research findings (8, 20).

### 2.3 Technical principles and workflow of dynamic navigation

#### 2.3.1 Preoperative planning and registration

High-resolution CBCT imaging is essential for DN, allowing for precise virtual planning of implant placement. Fiducial markers, placed in optimal anatomical locations, are used for accurate registration of the patient's anatomy with the navigation system (21). Proper marker placement also ensures maximum tracking accuracy and surgical precision (6, 7, 8).

#### 2.3.2 Intraoperative navigation and accuracy maintenance

During surgery, DN provides real-time visualization of drill trajectory and implant positioning, with continuous calibration checks to maintain system accuracy (6, 8). Mean angular deviation of only 2.05° and a coronal displacement of <2 mm, demonstrating DN's high accuracy in implant placement (9, 22, 23).

#### 2.3.3 Postoperative assessment and outcome validation

DN systems incorporate specialized software for postoperative verification, comparing planned vs. actual implant positions. Studies have validated DN's effectiveness in reducing implant misalignment and improving long-term stability (24). Superior clinical outcomes in DN-assisted procedures, with significantly higher implant survival rates over conventional approaches (6, 9, 25).

### 2.4 The DN-PUBLIC framework: A public health strategy for dynamic navigation integration

Evidence based and data informed implementations have been reported and proven to be the edifice of any framework and extrapolation of such findings into clinical practice, harnessing public potential (26). Dynamic Navigation (DN) delivers a transformative breakthrough to dental practice by enhancing implantology through advanced precision and improved safety and easier access to the procedure. The DN-PUBLIC framework gives healthcare systems worldwide an organised structure to integrate DN technology for maximum public health benefits. The expanded model supports the United Nations Sustainable Development Goals (SDGs) by enabling DN to deliver meaningful contributions to universal oral health improvement and healthcare equity and economic sustainability.

#### 2.4.1 Digital innovation: advancing precision through AI and technology

##### 2.4.1.1 Public health relevance

Enhances procedural safety and reduces surgical errors *(SDG 3: Good Health & Well-being)*.

Facilitates personalized medicine and precision dentistry *(SDG 9: Industry, Innovation, and Infrastructure)*.

Generates real-time, data-driven insights for quality improvement *(SDG 17: Partnerships for the Goals)*.

##### 2.4.1.2 Key advancements & future roadmap

AI-Powered Predictive Analytics: Enhancing DN systems with machine learning models to predict optimal implant placement, reducing human error and improving long-term success rates.

Automated Decision-Support Systems: Creating AI-driven tools that provide live feedback during procedures, ensuring superior outcomes.

Telemedicine & Remote DN Applications: Developing DN-compatible teledentistry solutions for underserved regions.

Ethical AI Integration: Establishing global regulatory standards to ensure the safe and fair application of AI-driven DN.

#### 2.4.2 Navigation training: standardizing education for skill development

##### 2.4.2.1 Public health relevance

Reduces reliance on specialized surgeons, expanding service accessibility *(SDG 4: Quality Education & SDG 10: Reduced Inequalities)*.

Enhances training for general practitioners and early-career dentists *(SDG 8: Decent Work and Economic Growth)*.

Promotes lifelong learning and continuing professional development (CPD).

##### 2.4.2.2 Key advancements & future roadmap

VR & AR-Based DN Simulations: Immersive training programs that allow clinicians to practice in a risk-free virtual environment.

Standardized DN Certification Programs: Establishing internationally recognized qualifications endorsed by leading dental organizations.

Integration into Public Health Systems: Embedding DN training into government-backed dental education initiatives.

Hands-On Training in Community Clinics: Expanding DN exposure through real-world practice in public healthcare settings.

#### 2.4.3 Patient-centered care: enhancing safety, comfort, and quality of life

##### 2.4.3.1 Public health relevance

Minimally invasive techniques reduce pain, anxiety, and complications *(SDG 3: Good Health & Well-being)*.

Improved adherence to dental care among anxious patients *(SDG 10: Reduced Inequalities)*.

Higher patient satisfaction and improved long-term oral health outcomes.

##### 2.4.3.2 Key advancements & future roadmap

Evidence-Based Guidelines for DN Surgery: Developing comprehensive clinical protocols to standardize care.

Longitudinal Studies on Patient-Reported Outcomes: Researching the psychosocial impact of DN-assisted procedures.

Accessibility for Vulnerable Populations: The implementation of DN into public dental programmes should expand to provide equal dental care access for all.

#### 2.4.4 Universal accessibility: bridging gaps in dental care equity

##### 2.4.4.1 Public health relevance

Breaks geographical and economic barriers to advanced dental care *(SDG 1: No Poverty & SDG 10: Reduced Inequalities)*.

Empowers general dentists to perform complex procedures with confidence *(SDG 4: Quality Education)*.

Supports remote areas through mobile DN-assisted clinics *(SDG 11: Sustainable Cities and Communities)*.

##### 2.4.4.2 Key advancements & future roadmap

Public-Private Partnerships: Encouraging collaborations between governments and technology companies to subsidize DN technology.

Government Funding for DN in Rural Clinics: Advocating for DN technology in low-income and marginalized communities.

Mobile DN Units for Remote Populations: Deploying DN-assisted services via mobile dental vans.

#### 2.4.5 Budget efficiency: reducing healthcare costs and improving resource utilization

##### 2.4.5.1 Public health relevance

Reduces surgical revisions and complications, minimizing healthcare expenditures *(SDG 3: Good Health & Well-being & SDG 12: Responsible Consumption and Production)*.

Improves clinic efficiency, reducing chair time and optimizing resources *(SDG 9: Industry, Innovation, and Infrastructure)*.

Enables cost-effective treatment models for public health systems.

##### 2.4.5.2 Key advancements & future roadmap

Cost-Benefit Analysis for Policymakers: Conducting studies on DN's economic impact.

Insurance Coverage for DN-Assisted Procedures: Lobbying for DN-based treatments in universal healthcare plans.

Efficient Material Use and Waste Reduction: Encouraging eco-friendly and sustainable DN practices.

#### 2.4.6 Long-term success: ensuring implant longevity and sustainability

##### 2.4.6.1 Public health relevance

The technology improves implant survival rates by lowering costly medical interventions for better health outcomes (SDG 3: Good Health & Well-being).

Minimizes material wastage and promotes sustainable practices in implantology *(SDG 12: Responsible Consumption and Production)*.

Supports sustainable oral healthcare systems with long-term planning.

##### 2.4.6.2 Key advancements & future roadmap

Global Implant Registries for DN Success Tracking: Establishing large-scale databases to monitor implant outcomes.

Predictive Failure Models: Using AI to forecast implant risks and prevent failures.

Sustainable Biocompatible Materials: Encouraging research into environmentally friendly biomaterials.

#### 2.4.7 Integration into policy: establishing a regulatory and ethical framework

##### 2.4.7.1 Public health relevance

Ensures ethical and responsible adoption of DN technology *(SDG 16: Peace, Justice, and Strong Institutions)*.

Establishes guidelines for safe and standardized DN use *(SDG 17: Partnerships for the Goals)*.

Encourages inclusion in national and international oral health policies.

##### 2.4.7.2 Key advancements & future roadmap

International Consensus on DN Best Practices: Developing globally recognized standards.

Advocacy for DN in Universal Dental Care Policies: Working with WHO and health ministries.

Regulatory Oversight for AI in DN: Establishing laws to govern AI-driven decision-making.

#### 2.4.8 Clinical research: strengthening the evidence base for Dn

##### 2.4.8.1 Public health relevance

Provides robust data to support widespread DN adoption *(SDG 9: Industry, Innovation, and Infrastructure & SDG 17: Partnerships for the Goals)*.

Encourages research collaborations and data-sharing.

Fosters continuous technological advancements.

##### 2.4.8.2 Key advancements & future roadmap

Large-Scale RCTs on DN Effectiveness: Conducting high-quality clinical trials to validate DN benefits.

Global Research Networks for DN Data Sharing: Encouraging open-access repositories.

Public & Private Funding for DN Innovation: Supporting academic and industry partnerships.

### 2.5 Key policy actions for global dynamic navigation (DN) adoption

DN must reach maximum public health impact through policy-based implementation strategies that receive support from governments along with educational institutions and healthcare systems. A detailed plan exists below for each sector which specifies particular actions alongside benefits and sustainability elements.

A strategic alignment of actionable steps, responsible implementers, real-world outcomes, and linked Sustainable Development Goals (SDGs) is represented in Table 1.

**Table 1:** Key policy actions for global dynamic navigation (DN) adoption.

Policy action	Implementer	Practical implementation	Expected outcome	SDG linked
Integrate DN into national oral health policies and clinical guidelines	Ministries of Health, National Dental Councils	Update dental practice guidelines; mandate DN use in complex surgeries in government hospitals	Widespread DN adoption in public sector; improved quality of care	SDG 3, SDG 10, SDG 16
Subsidize DN systems in public clinics and rural hospitals	Governments, Health Ministries, Local Authorities	Budget allocation through national health missions and rural health infrastructure schemes	Affordable access to advanced implantology for underserved populations	SDG 1, SDG 3, SDG 11
Embed DN training in dental school curricula and CPD modules	Dental Councils, Academic Institutions	Revise BDS/MDS syllabi; implement mandatory DN training hours in CPD credit system	Standardized DN competency among all practicing dentists	SDG 4, SDG 8
Establish DN residency, fellowship, and tele-mentoring programs	Universities, Private Hospitals, Dental Colleges	Launch dedicated DN training tracks; implement remote live surgery mentoring platforms	Skilled workforce capable of precision-guided implant procedures	SDG 9, SDG 10
Create international certification and accreditation standards for DN practice	Dental Federations, ISO, WHO Collaborating Centres	Develop global DN qualification framework; accredit institutions via audit-based reviews	Quality assurance and global standardization of DN practices	SDG 4, SDG 17
Public-private partnerships (PPP) for DN technology development and distribution	Governments, Dental Tech Companies, Innovation Hubs	Support local DN startups, incentivize joint ventures, establish R&D zones	Cost reduction, innovation boost, domestic manufacturing of DN systems	SDG 9, SDG 17
Conduct longitudinal, multi-center DN research and RCTs	Public Health Agencies, Universities, Research Councils	Fund competitive grants; set up national DN implant registries; enable multi-site data pooling	Evidence-based practice, real-world effectiveness data, global best practices	SDG 12, SDG 17

### 2.6 DN integration strategy table

A strategic plan for Digital Navigation (DN) implementation exists in this Table 2 which guides public health systems and dental education and clinical practise through governmental and educational and healthcare system partnerships.

**Table 2:** Dynamic navigation strategic intervention recommendation.

Stakeholder	Policy actions & implementation strategies	Public health impact	Sustainability measures
Governments & Policymakers	(a) Incorporate DN into national oral health strategies. (b) Allocate funding to subsidize DN in public institutions. (c) Implement regulatory frameworks for safety and quality. (d) Establish DN-focused tele-dentistry programs. (e) Support local DN system manufacturing via partnerships.	(a) Increases access to DN-supported treatments. (b) Reduces disparities by equipping general practitioners (GPs) with DN. (c) Optimizes resources and reduces implant failures. (d) Encourages government-backed DN research.	• Monitor adoption and outcomes via national surveillance. • Develop cost-benefit analyses for economic impact. • Incentivize public-private partnerships for DN advancement.
Educational Institutions	(a) Mandate DN training in undergraduate and postgraduate curricula. (b) Develop DN-specific residencies and fellowships. (c) Create standardized DN certification programs. (d) Integrate AI-powered DN simulation tools. (e) Incorporate tele-mentoring and global training modules.	(a) Enhances surgical precision and patient safety. (b) Increases competency and confidence of dental professionals. (c) Addresses geographic and skill-based disparities. (d) Facilitates global equity in dental innovation access.	• Monitor learning outcomes using competency-based tools. • Foster cross-institutional and international collaborations. • Build scalable, digital-first DN education platforms.
Healthcare Systems	(a) Require DN for complex procedures in public hospitals. (b) Expand DN use in community dental clinics. (c) Deploy DN-equipped mobile units in underserved areas. (d) Offer subsidized DN access for low-income populations. (e) Integrate AI-enhanced DN tools for diagnostics and planning.	(a) Lowers implant failure rates and post-op issues. (b) Reduces dependency on specialist referrals. (c) Improves surgical efficiency and public system capacity. (d) Optimizes healthcare workforce utilization.	• Define benchmarks and KPIs for DN integration. • Integrate DN within national reimbursement and insurance models. • Use DN-generated data for workforce planning and continuous improvement.

Barriers and Enablers to DN Integration in Public Health (Table 3).

**Table 3:** Key system-level challenges and targeted policy solutions for accelerating adoption.

Barrier	Description	Practical Policy Enabler
1. High initial cost of DN systems	Clinics and hospitals struggle to afford DN equipment due to capital expense.	• Government bulk procurement programmes should be established to decrease the cost per unit. • The government should establish low-interest loan programmes together with public-private partnership equipment leasing options. • The government should establish tax incentives together with GST relief programmes for DN equipment purchasing.
2. Insufficient training and skill development	General dentists and early-career practitioners lack exposure to DN systems.	• Mandate DN training in dental curricula (undergraduate/postgraduate). • Create digital DN simulation platforms for low-cost, scalable access. • Launch DN fellowships with practical modules in public hospitals.
3. Limited regulatory and clinical guidelines	Absence of structured policy on DN use and safety standards.	• The establishment of regulatory task forces operates at both national and regional levels. • Develop clinical protocols and consent guidelines for DN procedures. • Include DN modules in medico-legal and ethical training.
4. Inadequate infrastructure in rural and underserved areas	Poor digital connectivity, limited electricity, and lack of trained personnel.	• Deploy solar-powered mobile DN dental vans with offline-ready systems. • Train mid-level providers in DN-assisted workflows. • Use satellite connectivity for remote telementoring and navigation.
5. Lack of insurance/reimbursement coverage	DN-assisted procedures not covered under public or private insurance plans.	• Include DN within national insurance schemes (e.g., Ayushman Bharat). • Engage insurers and actuarial teams to quantify DN's cost-benefit. • Offer pilot reimbursements for DN to encourage insurer buy-in.

## 3 Conclusion: A sustainable future with DN in public health dentistry

Dynamic navigation represents a paradigm shift in implant dentistry, improving precision, reducing complications, and enhancing oral health outcomes from a public health perspective. By expanding access to high-quality implantology, minimizing surgical risks, and optimizing healthcare costs, DN contributes significantly to equitable oral healthcare delivery. The DN-PUBLIC framework aligns with global health priorities and the Sustainable Development Goals, ensuring Dynamic Navigation is integrated responsibly and equitably into public health dentistry. By fostering innovation, accessibility, policy integration, and economic sustainability, DN can reshape the future of oral healthcare, improving lives worldwide.
